# Atomic-scale disproportionation in amorphous silicon monoxide

**DOI:** 10.1038/ncomms11591

**Published:** 2016-05-13

**Authors:** Akihiko Hirata, Shinji Kohara, Toshihiro Asada, Masazumi Arao, Chihiro Yogi, Hideto Imai, Yongwen Tan, Takeshi Fujita, Mingwei Chen

**Affiliations:** 1 WPI Advanced Institute for Materials Research, Tohoku University, Sendai 980-8577, Japan; 2 Quantum Beam Unit, National Institute for Materials Science (NIMS), 1-1-1 Kouto, Sayo, Hyogo 679-5148, Japan; 3 Information Integrated Materials Research Unit, Research Center for Information Integrated Materials, NIMS, 1-2-1 Sengen, Tsukuba, Ibaraki 305-0047, Japan; 4 Division of Research & Utilization, Japan Synchrotron Radiation Research Institute, Hyogo 679-5198, Japan; 5 Schools of Materials Science, Japan Advanced Institute of Science and Technology, Nomi, Ishikawa 923-1291, Japan; 6 JST, PRESTO, 4-1-8 Honcho, Kawaguchi, Saitama 332-0012, Japan; 7 Device-functional Analysis Department, NISSAN ARC Ltd., 1 Natsushima, Yokosuka 237-0061, Japan; 8 State Key Laboratory of Metal Matrix Composites and School of Materials Science and Engineering, Shanghai Jiao Tong University, Shanghai 200030, China; 9 JST, CREST, 4-1-8 Honcho, Kawaguchi, Saitama 332-0012, Japan

## Abstract

Solid silicon monoxide is an amorphous material which has been commercialized for many functional applications. However, the amorphous structure of silicon monoxide is a long-standing question because of the uncommon valence state of silicon in the oxide. It has been deduced that amorphous silicon monoxide undergoes an unusual disproportionation by forming silicon- and silicon-dioxide-like regions. Nevertheless, the direct experimental observation is still missing. Here we report the amorphous structure characterized by angstrom-beam electron diffraction, supplemented by synchrotron X-ray scattering and computer simulations. In addition to the theoretically predicted amorphous silicon and silicon-dioxide clusters, suboxide-type tetrahedral coordinates are detected by angstrom-beam electron diffraction at silicon/silicon-dioxide interfaces, which provides compelling experimental evidence on the atomic-scale disproportionation of amorphous silicon monoxide. Eventually we develop a heterostructure model of the disproportionated silicon monoxide which well explains the distinctive structure and properties of the amorphous material.

## Abstract (web summary)

Amorphous silicon monoxide is known to undergo disproportionation to silicon- and silicon dioxide-like regions, however direct observation of the atomic-scale heterogeneity is still missing. Here, the authors use angstrom-beam electron diffraction to reveal precise structural details of this unusual material.

## Graphical abstract

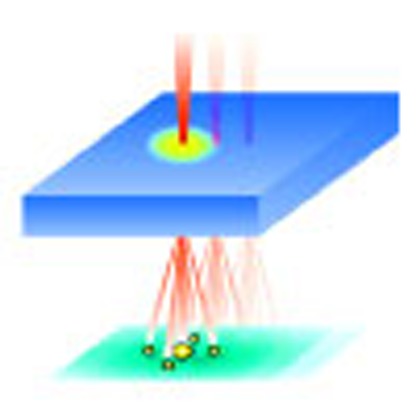

Silicon monoxide (SiO), the most common oxide of Si (ref. 1), was first reported by Charles Mabery^2^ and has been widely used as surface coatings, insulating layers in integrated circuits, dielectric material in capacitors and anode materials for Li-ion batteries^3^,^4^,^5^,^6^,^7^. However, since it was discovered, amorphous SiO has been the subject of much investigation and controversy^1^,^2^. In particular, the atomic structure of SiO has been debated for nearly a century despite numerous experimental and theoretical efforts devoted to this problem^8^,^9^,^10^,^11^,^12^. The uncommon +2 valence state of Si in the amorphous material cannot be described by either Si–4Si or Si–4O tetrahedra that have been successfully employed in the continuous random network models of amorphous Si and SiO_2_ (refs 13, 14, 15, 16, 17, 18, 19). Although a random bonding Si-(Si_4−*x*_O_*x*_) tetrahedral configuration can satisfy the requirements in Si valence state and stoichiometry of SiO (ref. 9), experiments and theoretical calculations have suggested that it is inherently unstable and undergoes an unusual disproportionation by forming amorphous Si- and SiO_2_-like clusters^8^,^12^. Local chemical analyses by transmission electron microscopy (TEM)^11^ and atom probe tomography^7^ have supported the existence of Si clusters in the Si–O matrix. However, it has been long known that the heat of combustion of amorphous SiO is significantly higher than that of an equilibrium mixture of amorphous Si and SiO_2_ (ref. 8) and, importantly, the X-ray diffraction (XRD) patterns of amorphous SiO cannot be interpreted by the summation of amorphous Si and SiO_2_ spectra^10^. Apparently, amorphous SiO is not a simple composite of amorphous Si and SiO_2_ clusters but may have a unique atomic structure, possibly, in the interfacial regions between Si and SiO_2_ domains as suggested by Hohl and co-authors^12^. Nevertheless, in spite of extensive investigations by XRD, X-ray photoelectron spectroscopy, X-ray Raman scattering, small-angle X-ray scattering and so on^12^,^20^,^21^,^22^, these techniques only provide average or spectroscopic information on the structure of the amorphous SiO. The unique and well-defined local atomic configurations of SiO have not been directly realized by experiments mainly because of the limitation in spatial resolution of conventional diffraction methods.

In this study we employ our recently developed angstrom-beam electron diffraction (ABED) method^23^,^24^ to investigate the local structure of amorphous SiO, which is supplemented by synchrotron high-energy XRD (HEXRD) and computational simulations based on molecular dynamics (MD) and reverse Monte Carlo (RMC) calculations. The ABED patterns from Si and SiO_2_-like nanoscale regions in amorphous SiO are obtained, as well as those from the Si/SiO_2_ interfaces. The diffraction intensity profile from the interfaces shows a unique feature different from either Si or SiO_2_. On the basis of ABED and HEXRD experiments, we develop a heterostructure model of the disproportionated amorphous SiO which well explains the structure and properties of the amorphous material.

## Results

### High-energy X-ray diffraction

**Figure 1:**
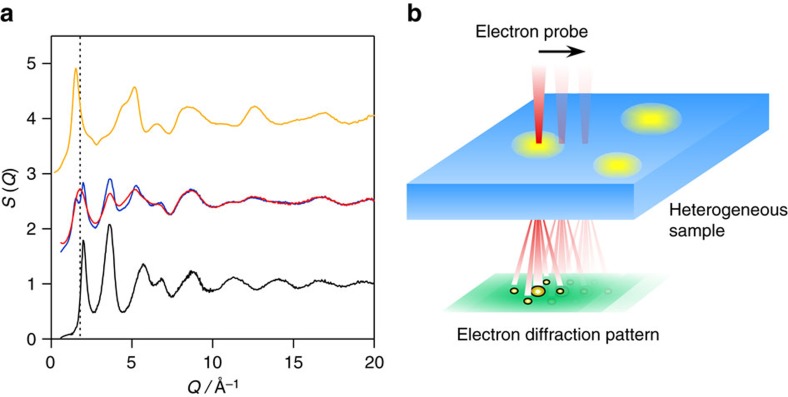
HEXRD and ABED experiments of amorphous SiO. (**a**) X-ray total structure factors *S*(*Q*) of amorphous SiO, Si and SiO_2_. The X-ray *S*(*Q*) of amorphous SiO obtained from HEXRD (red) is shown together with the *S*(*Q*) data of amorphous Si (black) and SiO_2_ (orange) reported in the refs 15 and 19^19^, respectively. The *S*(*Q*) profile for the summation of amorphous Si and SiO_2_ is shown as a blue curve. The dotted line is a guide to the eyes. The weighting factors of Si–Si, Si–O and O–O pairs for X-rays used in the RMC modelling are plotted in Supplementary Fig. 10, together with those for electrons. (**b**) The schematic diagram of the ABED measurements of amorphous SiO with nanoscale structural heterogeneity. Nano-regions indicated by yellow colour are structurally different from those indicated by blue colour. Diffraction patterns from yellow and blue nano-regions, and also their interface can be acquired by using ABED technique.

Commercially available amorphous SiO, fabricated by a vacuum sublimation, was used in this study (Osaka Titanium Technologies Co., Ltd.). Figure 1a shows the X-ray structure factor S(*Q*) of amorphous SiO (red curve) obtained by HEXRD, together with those of amorphous Si (black curve) and SiO_2_ (orange curve) reported in the literature^15^,^19^. The S(*Q*) of amorphous SiO appears to be intermediate between those of amorphous Si and SiO_2_. However, the summation curve (blue) of amorphous Si and SiO_2_ is not fully consistent with that of SiO, agreeing with previous observations^10^. In particular, the first sharp diffraction peak of the summation data splits into two sub-peaks, which cannot reproduce the first peak at *Q*∼1.8 Å^−1^ of amorphous SiO. The good agreement between the summation curve and SiO at the high *Q* portion indicates that the short-range structure of amorphous SiO could be similar to those of Si–4Si and Si–4O tetrahedra in amorphous Si and SiO_2_.

### Transmission electron microscopy

**Figure 2:**
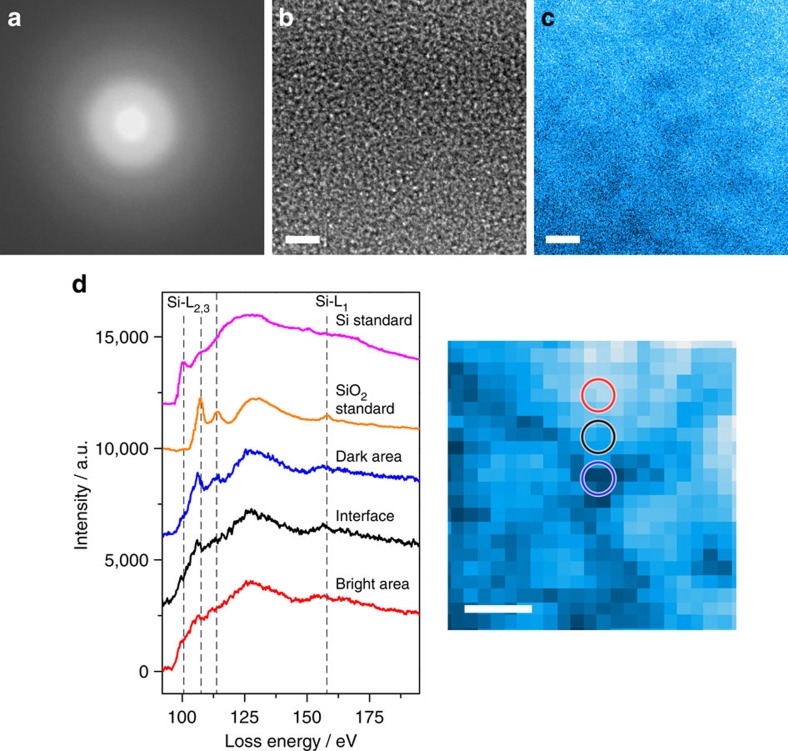
TEM/STEM micrographs and EELS spectra of amorphous SiO. (**a**) Selected area electron diffraction pattern; (**b**) High-resolution TEM image; and (**c**) HAADF-STEM image of the amorphous SiO. Scale bar, 2 nm. (**d**) EELS profiles (Si-*K* edge) taken from the dark (blue circle), bright (red circle) and interfacial (black circle) regions in the HAADF-STEM image. Standard EELS profiles of amorphous Si and SiO_2_ were extracted from the literature^11^. Scale bar, 2 nm.

The amorphous nature of SiO was verified by aberration-corrected TEM and selected area electron diffraction (Fig. 2a and b). The high-resolution TEM image shows typical maze-like contrast of amorphous materials. Neither a nanocrystalline phase nor inhomogeneity can be seen from the phase-contrast micrograph. On the other hand, the high-angle annular dark field scanning TEM (HAADF-STEM) image in Fig. 2c exhibits a faint inhomogeneous contrast with dark and bright nano-sized domains. Since HAADF-STEM is sensitive to the local density and chemistry, the contrast variation may result from the disproportionation of SiO. Separate STEM electron energy loss spectroscopy (EELS) spectra of Si-*L* edges taken from the dark, bright and interfacial regions in the HAADF-STEM image show that the Si bonding states in the dark and bright regions are different (Fig. 2d). The local EELS spectra for the dark regions are similar to those of amorphous SiO_2_ while the ones from the bright regions are analogous to amorphous Si. The EELS spectra of amorphous Si and SiO_2_ were taken from the standard samples for comparison^11^.

### Angstrom-beam electron diffraction

**Figure 3:**
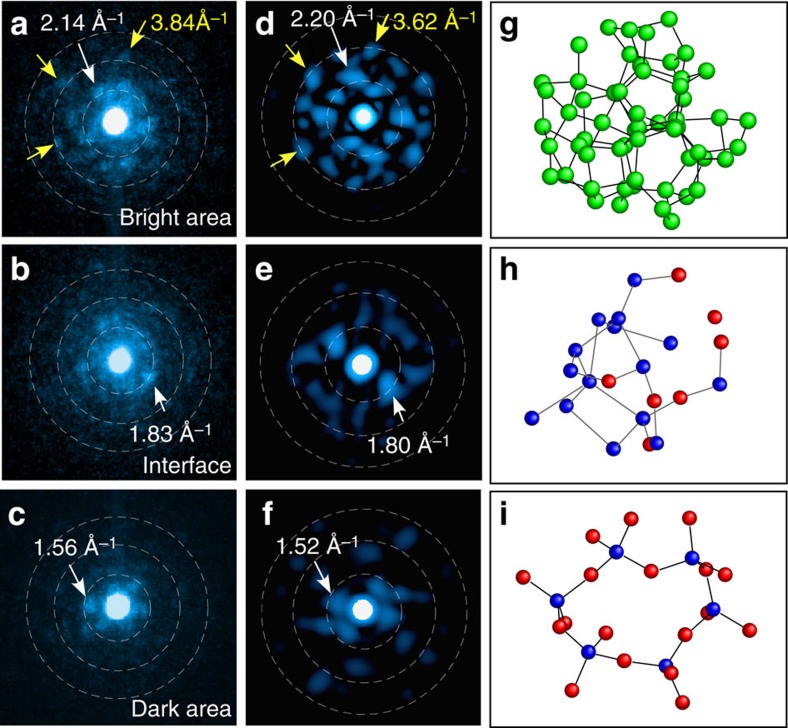
Experimental/simulated ABED from SiO local structures. (**a**–**c**) Typical ABED patterns taken from bright, interface and dark regions in the HAADF-STEM image shown in Fig. 2c. (**d**–**f**) The simulated ABED patterns based on the atomic models of amorphous Si, interfacial suboxide-type tetrahedra and amorphous SiO_2_ in **g**–**i**. The consistence between the experimental and simulated ABED patterns was achieved by systematically rotating the atomic models to proper orientations that agree with the experimental ABED patterns. The *Q* values as three dashed white rings shown in **a**–**c** are ∼1.8, 3.6 and 5.2 Å^−1^, which correspond to those of the first three peaks in X-ray total structure factor *S*(*Q*) of amorphous SiO shown in Fig. 1a. The atomic models shown in **g**–**i** were typical configurations found in the large models (MD model of pure amorphous Si (Supplementary Fig. 1a), MD–RMC model of amorphous SiO (Fig. 4a), MD model of pure amorphous SiO_2_ (Supplementary Fig. 1b) for **g**–**i**, respectively).

**Figure 4:**
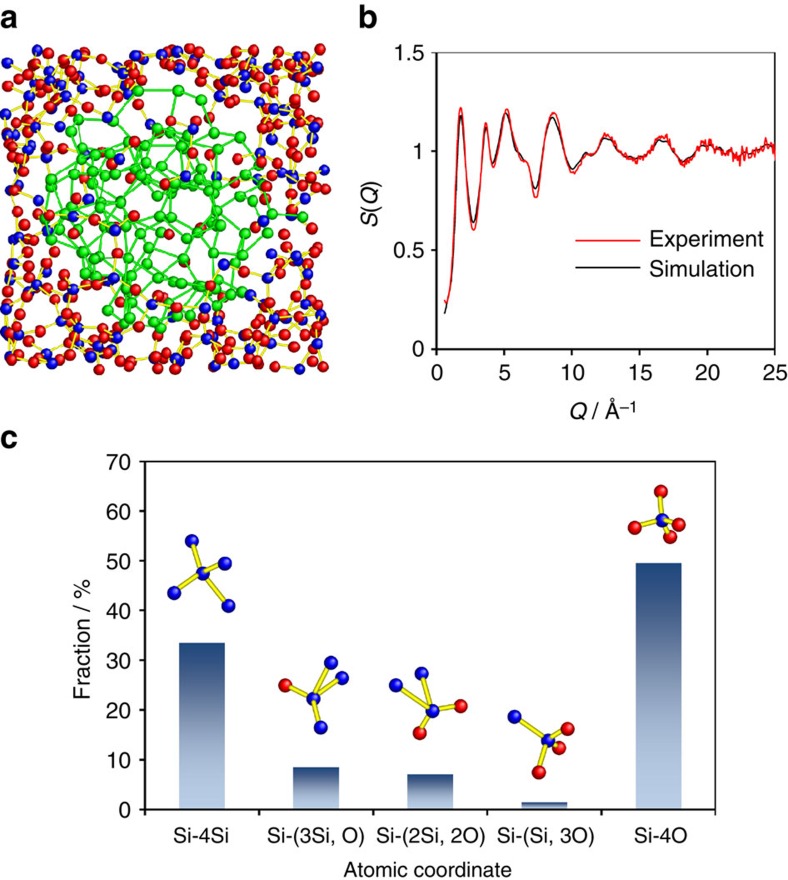
Atomic model of disproportionated amorphous SiO. (**a**) Reconstructed heterostructure model of amorphous SiO. The inner part corresponds to an amorphous Si cluster and the outer part is amorphous SiO_2_ matrix. The blue, red and green circles denote Si and O in amorphous SiO_2_ and Si in the Si cluster, respectively. (**b**) Experimental and simulated X-ray total structure factor *S*(*Q*) curves. The red curve denotes the experimental *S*(*Q*) data obtained from the HEXRD measurements. The black curve shows the *S*(*Q*) data obtained from heterostructure model after RMC refinement. (**c**) Fractions of the five atomic coordinates found in amorphous SiO. Si–4Si and Si–4O are from the Si cluster and SiO_2_ matrix while Si-(3Si, O) Si-(2Si, 2O) and Si-(Si, 3O) appear at the interfacial regions between the Si cluster and amorphous SiO_2_ matrix.

The local atomic structure of amorphous SiO was investigated by ABED as illustrated in Fig. 1b. The full width at half maximum electron probe was set as ∼0.8 nm to match the size of short-range order in the amorphous material. Different from conventional nano-beam electron diffraction, the convergence angle of the electron beam for ABED is as small as 1.0 mrad to form a nearly parallel electron probe. Figure 3a–c shows three typical ABED patterns obtained from dark, bright and interface regions. The dash lines marked in the ABED patterns represent Debye-Scherrer rings, corresponding to the major peaks found in *S*(*Q*) of amorphous SiO, measured by HEXRD and most ABED spots locate at the well-defined rings. Thus the diffraction data from global HEXRD and local ABED are qualitatively consistent with each other. On the basis of the *Q* values of each diffraction spot and the angles between the diffraction vectors, the corresponding local structures of those ABED patterns can be determined. It is found that the diffraction patterns in Fig. 3a and c are akin to those of amorphous Si and SiO_2_ with the smallest *Q* values close to the first sharp diffraction peaks of Si (*Q*∼2.0 Å^−1^) and SiO_2_ (*Q*∼1.5 Å^−1^) in HEXRD. For amorphous Si-like pattern, even the feature *Q* values close to the second peak of Si (*Q*∼3.6 Å^−1^) can be seen. In contrast, the ABED patterns taken from the interface region (Fig. 3b) cannot be interpreted by either amorphous Si or SiO_2_. Interestingly, the smallest *Q* values (∼1.8 Å^−1^) in the ABED patterns are very close to the first sharp diffraction peak of SiO (*Q*∼1.8 Å^−1^). It is worth noting that the first sharp diffraction peak of SiO is in the middle between the Si (*Q*∼2.0 Å^−1^) and SiO_2_ (*Q*∼1.5 Å^−1^). This indicates that the interface regions may be suboxide SiO_x_ with a variety of atomic coordinates between Si–4Si and Si–4O. Indeed, the simulated ABED patterns based on suboxide-type tetrahedra match well with the experimental one. The simulated ABED patterns of amorphous Si, SiO_x_ and SiO_2_ clusters are shown in Fig. 3d–f, together with the corresponding atomic models (Fig. 3g–i), which are the typical configurations found in the large atomic models (MD model of pure amorphous Si (Supplementary Fig. 1a), MD–RMC model of amorphous SiO (Fig. 4a) and MD model of pure amorphous SiO_2_ (Supplementary Fig. 1b)). Although the atomic arrangements of amorphous SiO are highly disordered, symmetric patterns can be frequently obtained by ABED experiments and by rotating the structural models of the amorphous clusters to match the experimental results.

**Figure 5:**
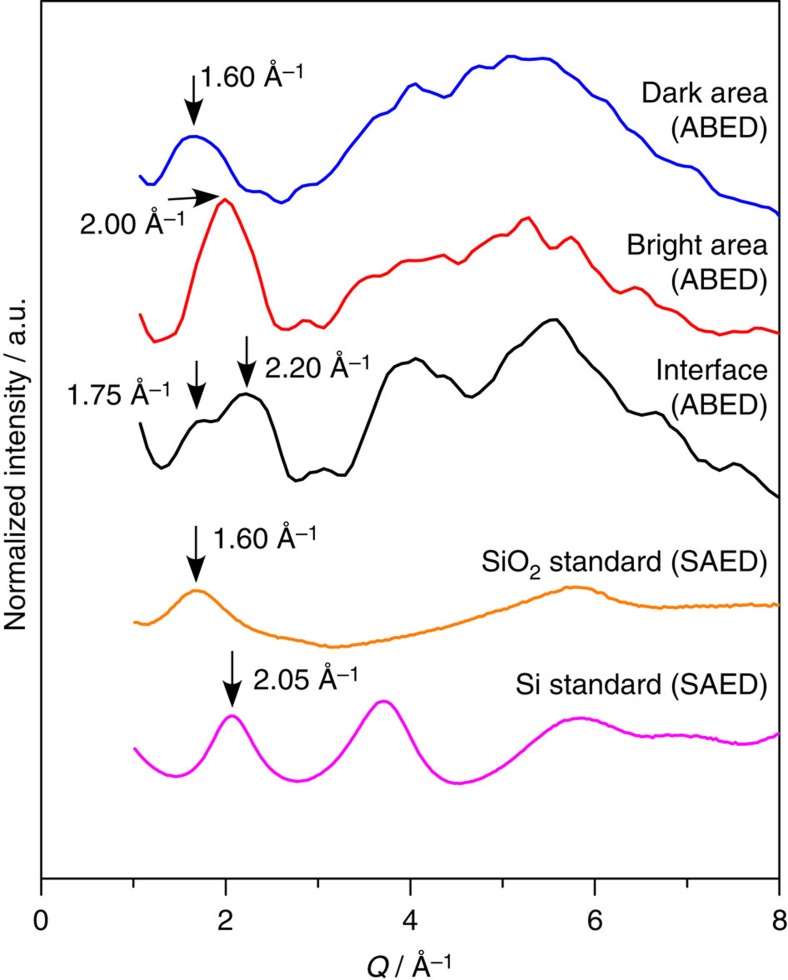
Integrated intensity profiles of ABED from different domains. The normalized intensity profiles were constructed by integrating 26, 35 and 14 ABED patterns obtained from dark, bright, and interface areas in HAADF-STEM images, respectively. The detailed procedure is described in Supplementary Fig. 2. For comparison, the selected area electron diffraction (SAED) profiles of amorphous SiO_2_ and Si are also shown here.

While it is a powerful approach to determine the local structure of amorphous SiO by carefully analysing individual ABED patterns as shown in Fig. 3, this method may only offer qualitative structure information for highly disordered and heterogeneous materials, such as amorphous SiO because of a large number of possible local atomic configurations. To overcome this shortage of ABED, we selectively integrated ABED patterns taken from different regions (dark, bright and interface), respectively, and transferred the two-dimensional diffraction pattern into intensity profiles to expose the structure of these heterogeneous domains in a statistic manner (Supplementary Fig. 2). Apparently, the normalized intensity profiles of ABED from the dark, bright and interface regions are observably different (Fig. 5). In fact, the profiles from dark and bright regions are similar to the normalized intensity profiles of amorphous SiO_2_ and Si obtained by selected area electron diffraction while the one from the interface region has unique diffraction features which the amorphous Si and SiO_2_ do not have. The statistical analysis further demonstrates the nanoscale disproportionation of amorphous SiO by forming amorphous Si and SiO_2_-like clusters as well as unique interface domains.

### Structural modelling

On the basis of the ABED and HEXRD results, we constructed an atomic model of amorphous SiO by the combination of MD simulations and RMC modelling. In this approach, the structure of homogeneous amorphous Si and amorphous SiO_2_ were first generated by MD simulations and verified by ABED (Fig. 3) and HEXRD. The heterogeneous structure revealed by ABED and HAADF-STEM was constructed by embedding an amorphous Si cluster into the amorphous SiO_2_ surrounding (Fig. 4a). The heterostructure was relaxed to reduce the total energy by MD simulations using variable-charge atomic potentials^25^,^26^. The relaxed model was further fitted to the experimental structure factor *S*(*Q*) by RMC modelling (Fig. 4b and Supplementary Fig. 3)^27^. The final structure model is shown in Fig. 4a, in which the coordinates of Si–4Si and Si–4O tetrahedral from the amorphous Si and SiO_2_ regions are still visible. Additionally, the suboxide-type tetrahedral coordinates (Si-(3Si, O), Si-(2Si, 2O) and Si-(Si, 3O)) revealed by ABED are formed at the Si/SiO_2_ interface regions during the structure relaxation (Supplementary Fig. 4). We also calculated the effective charges using Voronoi prescription in which atomic cells and volumes are based on geometry and the corresponding charges are calculated from the electron density enclosed inside the cell^28^,^29^. The electron density of the model was calculated using density functional theory (DFT). The electron charge of Si should be 0 and +4 in pure Si and SiO_2_ regions, respectively. In the interface regions between Si and SiO_2_, on the other hand, the charge of Si varies in a range from 0 to +4, depending on the surrounding atoms. The calculations confirm the presence of the suboxide-type tetrahedra in the interface regions (Supplementary Fig. 5). These suboxide-type tetrahedra keep the random networks of amorphous Si and SiO_2_ continuously across the interface regions. The number fractions of the five typical atomic coordinates are shown in Fig. 4c. There are considerable amounts of suboxide-type tetrahedra of Si-(3Si, O), Si-(2Si, 2O) and Si-(Si, 3O) from interface regions. These suboxide-type tetrahedra as the transition layers bridge amorphous Si and amorphous SiO_2_ by preserving the continuity of the random networks of Si and SiO_2_ (Supplementary Fig. 4). Therefore, the formation of the conspicuous interface regions with distinctive suboxide Si–O coordinates appear to be the structural origins of the amorphous SiO different from the simple mixture of amorphous Si and SiO_2_ in the thermal properties and X-ray diffraction.

### Raman spectroscopy and XANES

We further conducted Raman spectroscopy and X-ray absorption near-edge structure (XANES) analyses to obtain vibrational and electronic features of the amorphous SiO. In the Raman spectrum of amorphous SiO (Supplementary Fig. 6), besides the vibration bands similar to those of amorphous Si and SiO_2_, there are two characteristic peaks at ca. 510 and 645 cm^−1^. Although further theoretical analysis is required to explain the origins of the bands, based on the local diffraction data achieved in this study, it is most likely that the characteristic Raman bands are the suboxide-type vibration modes from the interfacial regions. Moreover, we measured XANES spectra of as-prepared and annealed SiO specimens (Supplementary Fig. 7). Annealing especially at 1050 °C completely led to a formation of (Si+SiO_2_) two phases, because the XANES spectrum can be understood as a simple summation of those of Si and SiO_2_. On the other hand, the spectrum of as-formed amorphous SiO is quite different from the summation. The unique peak ∼1843.5 eV for amorphous SiO found in between the peaks from Si and SiO_2_ is presumably due to the suboxide-type tetrahedra at the interface. This result is consistent well with the local EELS experiment shown in Fig. 2.

## Discussion

The fundamental challenge in structural characterization of disordered materials is that structural models with different heterogeneities can yield almost identical structure factors because X-ray and neutron diffractions only provide information about averaged structure. Indeed, one can construct a homogeneous model of amorphous SiO with basic units of fourfold silicon (total coordination number of silicon and/or oxygen atoms around a silicon atom is four) by RMC modelling (Supplementary Fig. 8), which is similar to a theoretical homogeneous model reported recently^30^. In principle, RMC modelling can fit any given diffraction data by introducing highly disordered uniform structures although these uniform configurations may not be thermodynamically realistic^31^. In contrast, it is difficult to construct an inhomogeneous amorphous structure only using conventional RMC modelling or MD simulations. In this study, we developed a new approach to construct an amorphous heterostructure model with the appropriate degree of inhomogeneity (∼1–2 nm) based on very local ABED and the combination of variable-charge MD simulation and RMC modelling. The experiment-based atomic structure can fully reproduce the experimental *S*(*Q*) obtained from the HEXRD measurements in all *Q* space. Moreover, DFT calculations demonstrate that the structural model of the amorphous SiO is energetically more stable in comparison with the homogeneous atomic model predicted by RMC and the composite model of amorphous Si and SiO_2_ constructed only by MD (Supplementary Fig. 9). Although the RMC approach, which is known to fail in determining definite local atomic structure, is also used to generate the initial structure of amorphous SiO, the uncertainness of the RMC can be overcome by the successful utilization of ABED which provides well-defined local atomic configurations in the heterogeneous SiO. Therefore, the experiment-based MD–RMC approach developed in this study could be a powerful and generic method to model disordered materials with structural heterogeneity based on both local and global diffraction data.

In summary, we systematically investigated the atomic structure of amorphous SiO by utilizing state-of-the-art ABED, complemented with synchrotron HEXRD and MD-RMC simulations. The ABED experiment provides direct evidence on the atomic-scale disproportionation in amorphous SiO, predicted by theoretical calculations. The distinctive interfacial structure between amorphous Si and SiO_2_ clusters, revealed by the sub-nanoscale electron diffraction, uncovers the structural origins of amorphous SiO, different from the simple mixture of Si and SiO_2_. The heterostructure model of disproportionated amorphous SiO resolves the long-standing question on the structure of amorphous SiO. Moreover, the MD–RMC procedure based on both local and global diffraction could be a generic approach to model atomic structure of inhomogeneous amorphous materials.

## Methods

### Angstrom-beam electron diffraction

We employed a JEOL JEM-2100F TEM/STEM system with double Cs-correctors (operated at 200 kV) for the measurements. All the ABED patterns were recorded with a charge-coupled device camera (Gatan, US1000). A coherent electron beam was produced using a specially designed small-condenser aperture with a diameter of 3.5 μm (Daiwa Techno Systems Co., Ltd.). The convergence angle was estimated to be 1.0 mrad. The instrumental parameters such as the spherical aberration coefficient, defocus and astigmatism were precisely measured using the Ronchigram method^32^. By using a scanning function of the STEM system, we were able to obtain a large number of ABED patterns (more than 10,000 frames) from a thin area near the specimen edge. The specimens with a thickness <5 nm were prepared by the conventional crushing method for the TEM/STEM observations. The ABED patterns were calculated using the multislice simulation software, which has been detailed in ref. 33.

### High-energy X-ray diffraction

The HEXRD measurements were performed on powder samples at room temperature using the BL04B2 beamline of SPring-8 (ref. 34). A two-axis diffractometer dedicated to the study of glass, liquids and amorphous materials was employed to measure the diffraction spectra. The energy of the incident X-rays was 61.4 keV and the diffraction patterns were measured in the transmission geometry. The intensity of the incident X-rays was monitored in an ionization chamber filled with Ar gas and the scattered X-rays were detected using three CdTe detectors. A vacuum chamber was used to suppress air scattering around the sample. The collected data were corrected using a standard programme^34^.

### Computational simulations

The models of amorphous Si and SiO_2_ were constructed by using MD methods (Supplementary Fig. 1). Three-body Tersoff-type^16^,^35^ and two-body Born–Mayer–Huggins-type^36^ atomic potentials were employed for amorphous Si and SiO_2_, respectively. The MD simulations were performed under constant-NVT conditions. First, 1,000 Si atoms for amorphous Si and 216 Si, and 432 O atoms for SiO_2_ were placed randomly in cubic MD cells with periodic boundary conditions. The densities used for amorphous Si and SiO_2_ were 2.33 and 2.30 g cm^−3^, respectively. To construct the model of SiO, we embedded a spherical amorphous Si cluster into the SiO_2_ matrix with a density of 2.15 g cm^−3^ and then relaxed at 1,000 K for short periods (5–100 fs) using variable-charge potentials where the electron charges are variable based on the atomic coordination environments^25^,^26^. The fractions of amorphous Si and SiO_2_ in the initial configuration, containing 128 Si atoms of amorphous Si as well as 128 Si and 256 O atoms for amorphous SiO_2_, were determined by the stoichiometry of SiO.

The RMC refinement was performed using the RMC++ code^37^. The atomic configuration produced by the MD simulation was employed as the starting structure. During the simulation based on the X-ray total structure factor, *S*(*Q*), the coordination number distribution of oxygen around silicon and that of silicon around silicon, as well as the bond angle distribution of O–Si–O, were constrained to the values obtained from the MD simulation to avoid producing unreasonable disordered local structure.

A simulation box containing 552 atoms obtained by MD and MD-RMC simulations on heterostructure models, and by RMC homogeneous modelling, were used for DFT calculations. The CP2K programme was used in the DFT mode. CP2K employs two representations of the electron density: localized Gaussian and plane wave basis sets. For the Gaussian-based (localized) expansion of the Kohn–Sham orbitals, we used a library of contracted molecularly optimized valence double-zeta plus polarization basis sets^38^, and the complementary plane wave basis set had a cutoff of 400 Rydberg for the electron density. The valence electron–ion interaction was based on the norm-conserving and separable pseudopotentials of the analytical form derived by Goedecker, Teter and Hutter^39^, and the generalized gradient corrected approximation of Perdew, Burke and Ernzerhof was adopted for the exchange-correlation energy function^40^.

### Data availability

The authors declare that all relevant data supporting the findings of this study are available from the authors on request.

## Additional information

**How to cite this article:** Hirata, A. *et al*. Atomic-scale disproportionation in amorphous silicon monoxide. *Nat. Commun.* 7:11591 doi: 10.1038/ncomms11591 (2016).

## Supplementary Material

Supplementary Information Supplementary Figures 1-10 and Supplementary References
