# Supplementary material for: Atomic-scale disproportionation in amorphous silicon monoxide
Source: Nat Commun. 2016 May 13;7:11591. doi: 10.1038/ncomms11591 (PMC4869258; doi:10.1038/ncomms11591)
Supplement: Supplementary Information — Supplementary Figures 1-10 and Supplementary References [file ncomms11591-s1.pdf]

Supplementary Figures

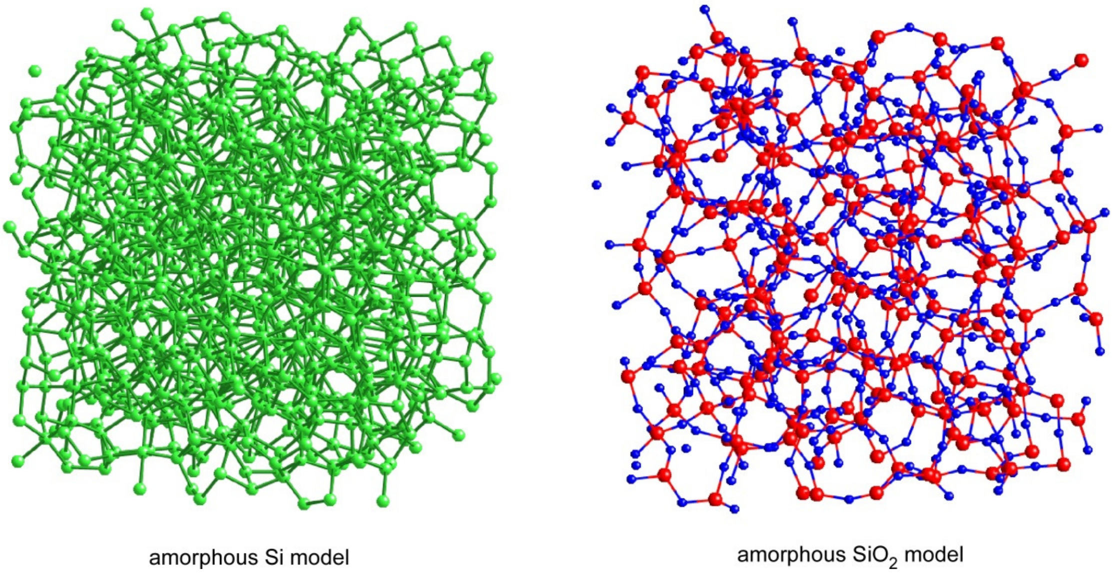

Supplementary Figure 1 Atomic configurations of amorphous Si and amorphous SiO_2 obtained by MD simulations.

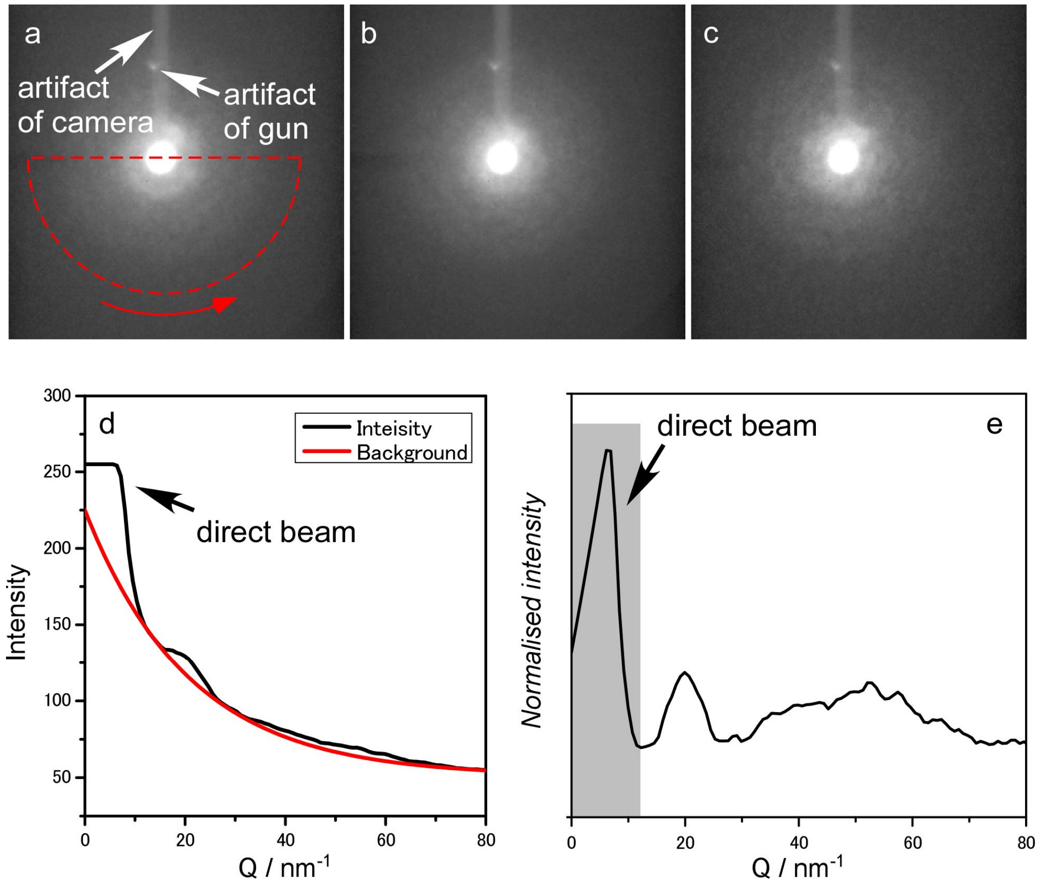

Supplementary Figure 2 Raw data of integrated ABED patterns obtained from **a**, bright areas (Si-like), **b**, dark areas (SiO_2-like), and **c**, gray areas (interface) in HAADF-STEM images. We used 26, 35, and 14 ABED patterns to construct the integrated patterns of **a**, **b**, and **c**, respectively. To remove artificial effects from electron gun and camera, we used the lower half part of patterns when getting integrated intensity profiles. Total 10 intensity line profiles were obtained from the lower half part and then averaged. We calculated normalised intensity curves from the averaged intensity profiles as shown in **d**. The background curves used here, which are an exponential function and similar to atomic scattering factors, are determined in an ad hoc manner simply to enhance intensity peaks. The peak top positions are basically unchanged by altering a gradient of the background curve. Finally we obtained normalised intensity profiles shown in **e** and remove an artificial peak which originates from the direct beam.

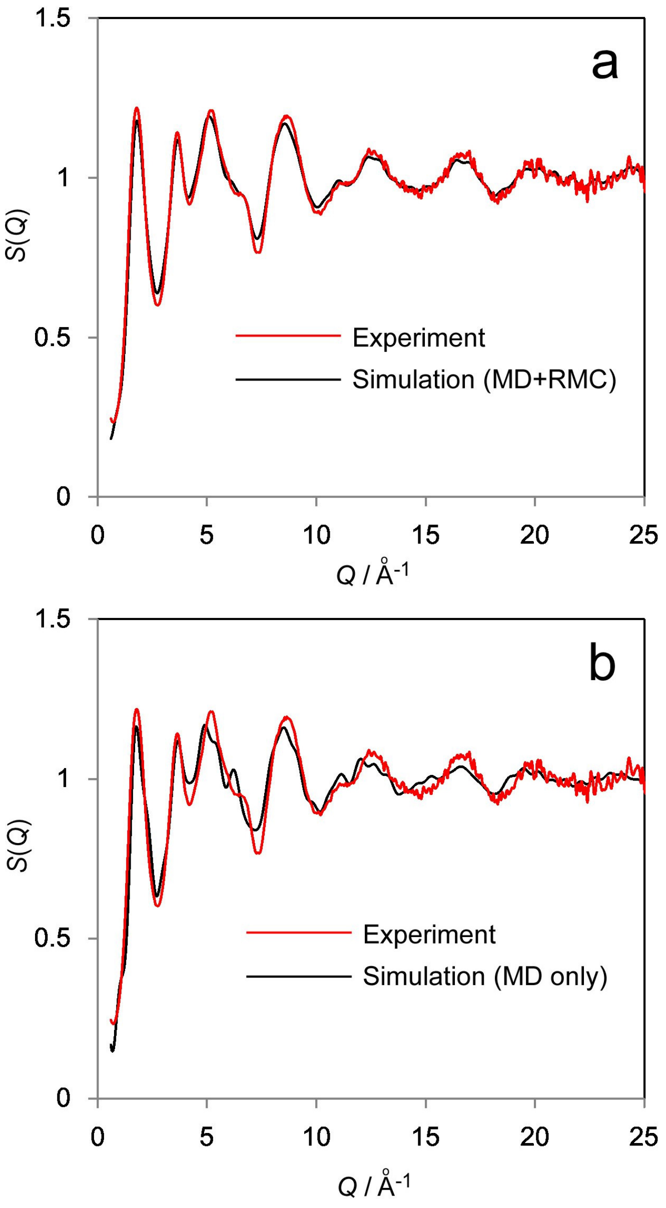

Supplementary Figure 3 **a**, Calculated X-ray total structure factor $S(Q)$ obtained from the final model and **b**, calculated $S(Q)$ obtained from the MD simulation, together with the experimental data.

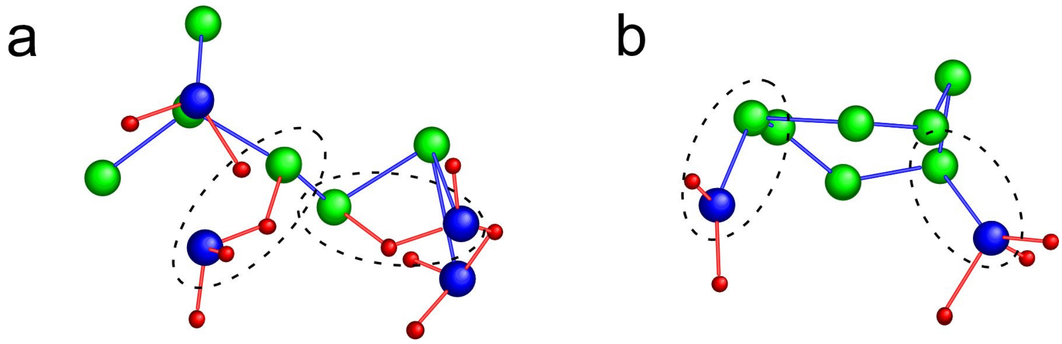

Supplementary Figure 4 Atomic configurations at the interface between Si and SiO_2 regions in the model of **Figure 4a**. **a**, Silicon atoms in amorphous Si region are bonded with silicon atoms in SiO_2 region via oxygen. **b**, Silicon atoms in amorphous Si region are bonded with silicon atoms in SiO_2 region. Red, blue, and green denote oxygen, silicon in SiO_2 region, and silicon in Si region, respectively.

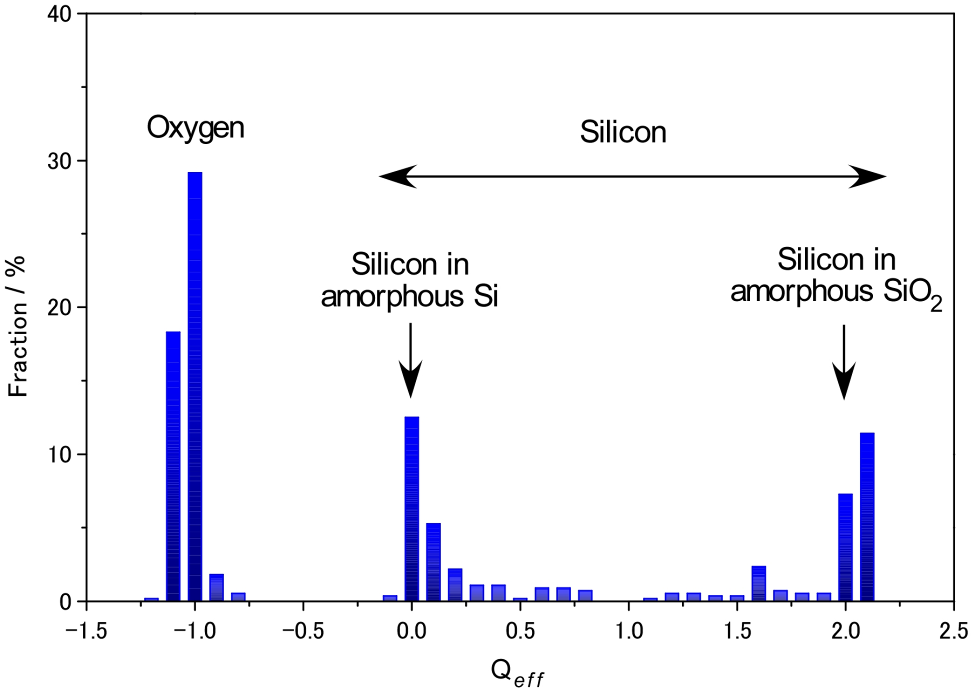

-

Supplementary Figure 5 The effective Voronoi charges (Q_{eff}) for silicon and oxygen atoms in the MD-RMC model. The electron density of the model was obtained by DFT calculation. Voronoi prescription of atomic volume is analogous to the Wigner-Seitz cell in crystalline materials. The silicon atoms with Q_{eff} between 0 and 2 can be assigned to silicon in suboxide-type tetarahedra in the interfacial regions. Voronoi prescription of atomic cell/volume is based on geometry, and the corresponding charge is calculated from the electron density enclosed inside the cell [refs. 1, 2].

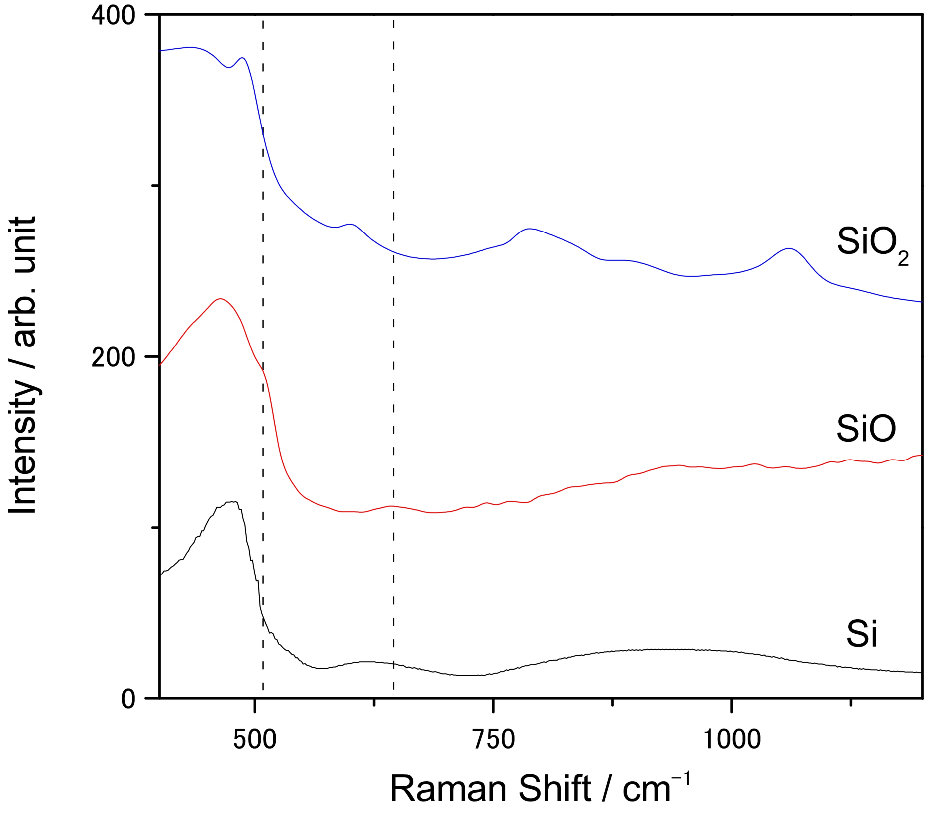

Supplementary Figure 6 Raman spectra of amorphous Si, SiO, and SiO_2. The data for amorphous Si was taken from the literature [ref. 3]. The characteristic peaks observed at 510 and 645 cm^{-1} for amorphous SiO are indicated by dashed lines. The Raman spectroscopy measurements were carried out by using a Renishaw Raman microscope (Renishaw InVia RM 1000) with an incident laser wavelength of 514.5 nm.

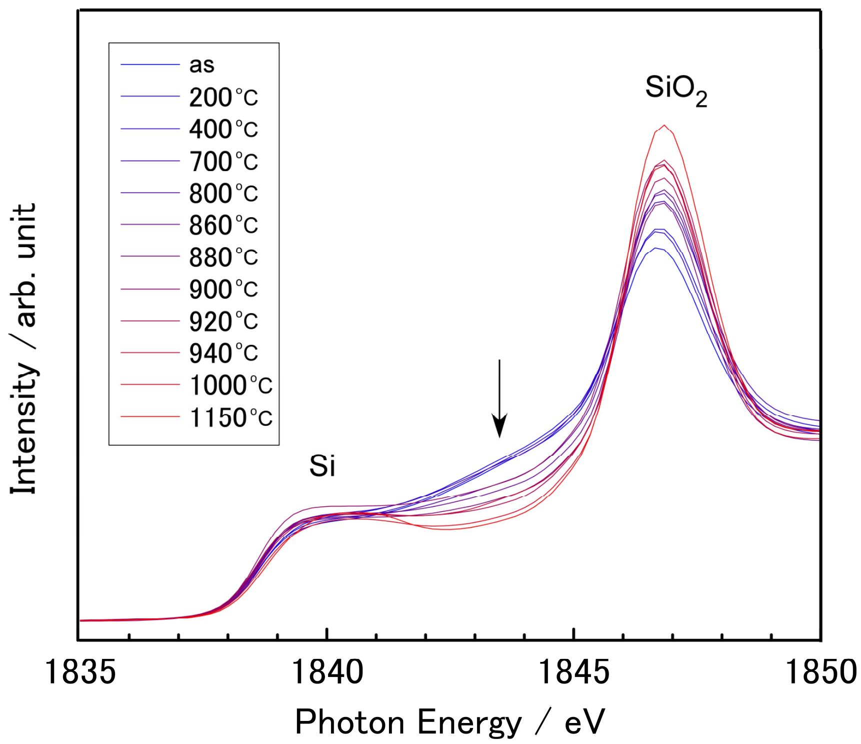

Supplementary Figure 7 The Si K-edge X-ray absorption near-edge structure (XANES) spectra of as-prepared and annealed SiO samples. The amorphous SiO was completely decomposed into ($Si+SiO_2$) two phases by annealing at 1150 °C. The peaks at about 1840 and 1847 eV originate from pure Si and SiO_2, respectively. The broad shoulder around 1843.5 eV indicated by the arrow is a characteristic feature of amorphous SiO. The XANES analyses were performed with the BL-10 beamline at the SR Centre of Ritsumeikan University. The spectra were acquired by using the total electron yield method.

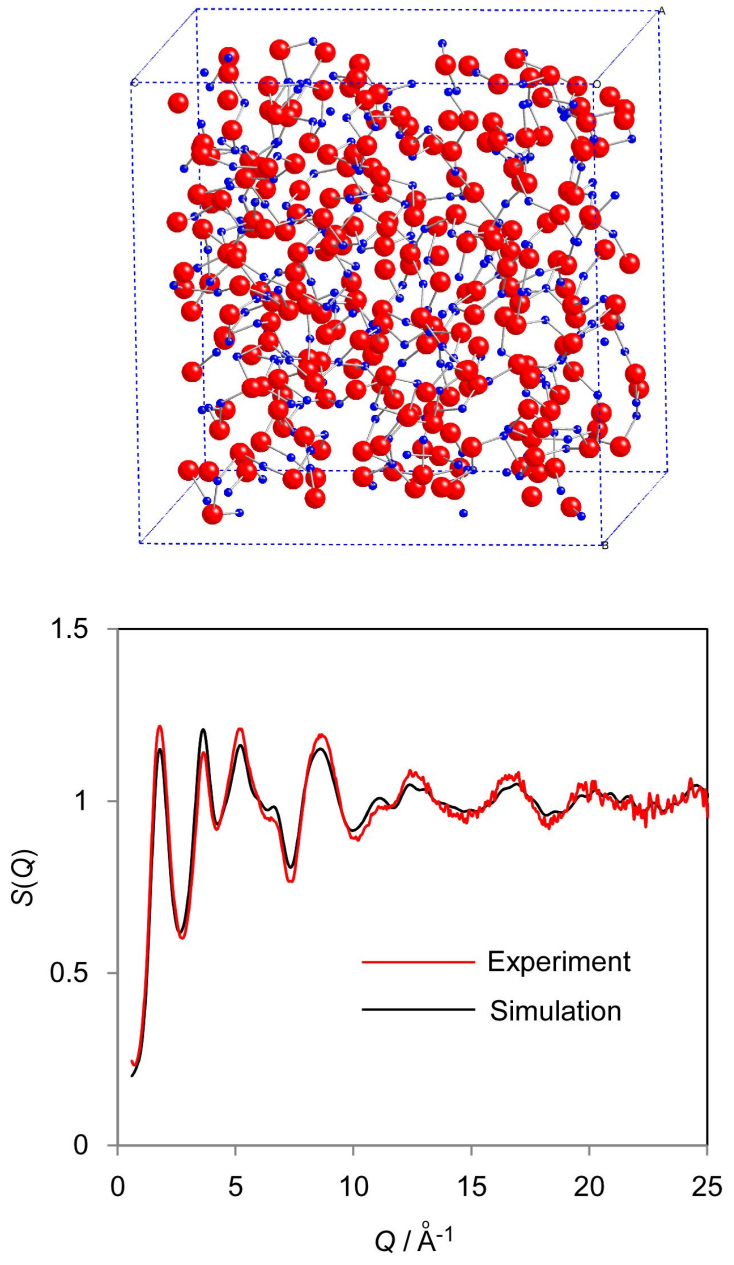

Supplementary Figure 8 Homogeneous model of amorphous SiO obtained by a reverse Monte Carlo modelling. Red and blue circles denote oxide and silicon atoms, respectively.

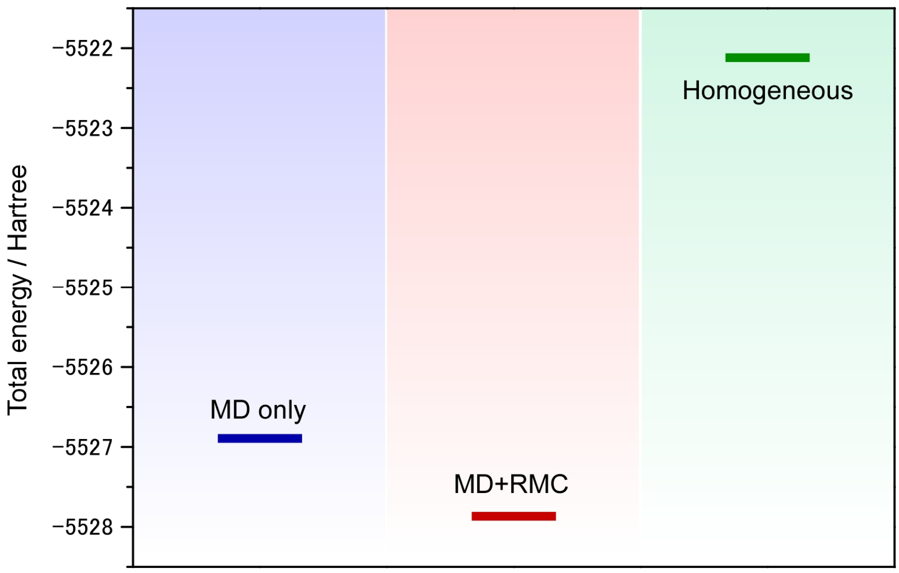

Supplementary Figure 9 DFT calculations of the total energies for composite models constructed by MD and MD-RMC simulations, together with the homogeneous model constructed by RMC.

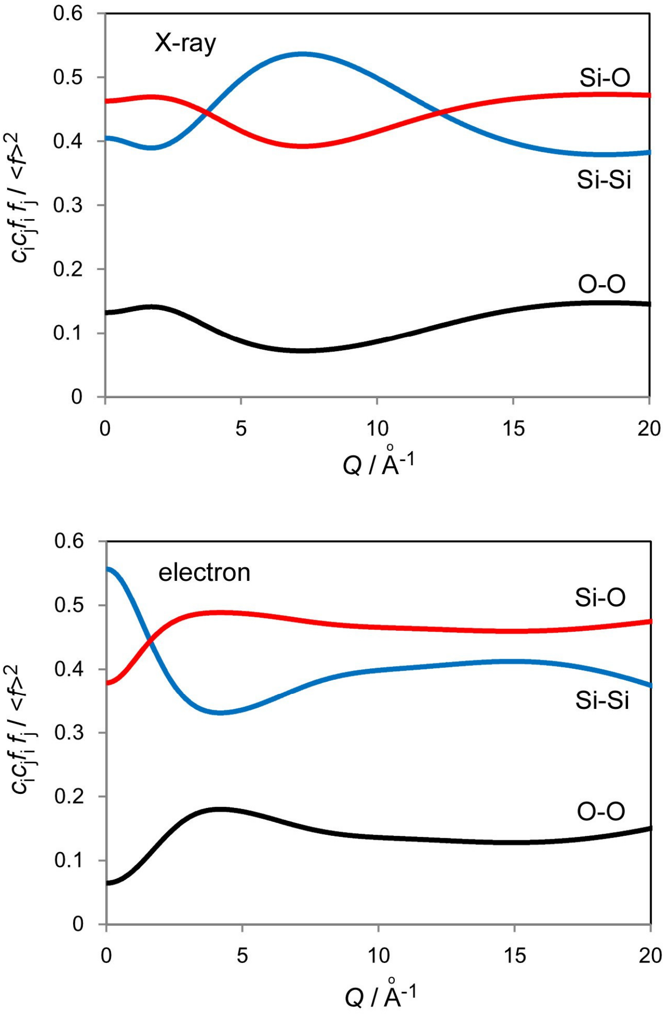

Supplementary Figure 10 Q dependent weighting factors ($c_i c_j f_i f_j$ / $<f>^2$) for X-ray and electron scattering. c_i and f_i denote atomic fraction and atomic scattering factor for i component, respectively. The curves for Si-O, Si-Si, and O-O pairs are plotted.

Supplementary References

1. Bickelhaupt, F. M., van Eikema Hommes, N. J. R., Guerra C. F., and Baerends, E. J., The Carbon-Lithium Electron Pair Bond in $(CH_3Li)_n$ (n = 1, 2, 4), *Organometallics* **15**, 2923-2931 (1996).
2. Guerra, C. F., Handgraaf, J. W., Baerends, E. J., and Bickelhaupt, F. M., Voronoi Deformation Density (VDD) Charges: Assessment of the Mulliken, Bader, Hirshfeld, Weinhold, and VDD Methods for Charge Analysis, *J Comput. Chem*. **25**, 189-210, (2004).
3. Brodsky, M. H., Cardona, M., & Cuomo, J. J., Infrared and Raman spectra of the silicon-hydrogen bonds in amorphous silicon prepared by glow discharge and sputtering, *Phys. Rev. B* **16**, 3556-3571 (1977)
